# Effect of Glass and Recycled Concrete Aggregate Content on Slag-Rich Alkali-Activated Concrete Reinforced with Tire-Derived Textile Fibers

**DOI:** 10.3390/polym18121470

**Published:** 2026-06-11

**Authors:** Ali Mardani, Metin İlhan, Hatice Gizem Şahin

**Affiliations:** Materials Technologies and 3D Concrete Printing R&D Laboratory, Department of Civil Engineering, Engineering Faculty, Bursa Uludag University, Bursa 16059, Türkiye; alimardani@uludag.edu.tr (A.M.); haticegizemsahin@gmail.com (H.G.Ş.)

**Keywords:** waste glass aggregate, recycled fiber, recycled concrete aggregate (RCA), tire-derived textile fiber (TDTF), alkali-activated concrete, geopolymer concrete

## Abstract

In this study, the effect of substituting waste glass aggregate and recycled concrete aggregate (RCA) at different ratios (20%, 40%, 60%, 80%, 100%) on the compressive strength performance of geopolymer concretes reinforced with tire-derived textile fibers (TDTF) was investigated. A total of 22 different mixtures were prepared, and their 7-day and 28-day compressive strengths, water absorption rates, and ultrasonic pulse velocity (UPV) were determined. The results showed that TDTF improved compressive strength in both waste aggregate series, with a more pronounced contribution at 28 days. Increasing the waste glass aggregate content reduced 28-day compressive strength by 16–31% compared with the control mixture, whereas RCA mixtures showed only 1–4% strength loss up to 60% replacement and 17–19% loss at higher replacement levels. Glass aggregate mixtures generally exhibited higher early-age strength, while RCA mixtures performed better at 28 days. TDTF addition increased the 28-day compressive strength by approximately 25–30%, depending on aggregate type and replacement level. The lowest water absorption value was obtained in the fiber-reinforced glass aggregate series, whereas the highest value was measured in the RCA series, mainly due to the porous adhered mortar on RCA particles. Based on the compressive strength, water absorption, and UPV results, RCA replacement levels up to 60% and glass aggregate replacement levels of 40–60% may be considered suitable for the mixtures examined in this study.

## 1. Introduction

Concrete is the most heavily consumed construction material in human history, with an annual global production exceeding approximately 30 billion tons. Such large-scale production brings two fundamental environmental problems: the rapid depletion of natural aggregate resources and high amounts of carbon dioxide released into the atmosphere due to Portland cement production. Portland cement production is held responsible for approximately 7–8% of global CO_2_ emissions, clearly demonstrating the construction sector’s direct share in climate change [1,2,3,4,5,6]. For this reason, over the last two decades, two main strategies have been emphasized to reduce the environmental burden of the cement industry: first, the development of alternative binder systems where Portland cement can be partially or completely substituted; and second, the widespread use of waste or recycled materials instead of natural aggregates [7,8].

Among low-cement and cement-free binder systems, alkali-activated materials have received considerable attention. Depending on precursor chemistry, these systems may include low-calcium aluminosilicate geopolymers as well as calcium-rich alkali-activated slag binders. The binder used in the present study is mainly based on GGBFS, with silica fume used as a supplementary silica-rich component and is activated with NaOH and Na_2_SiO_3_. Therefore, it is more accurately described as a slag-rich alkali-activated binder rather than a true low-calcium geopolymer. In such calcium-rich systems, the reaction products are generally associated with calcium aluminosilicate hydrate-type gels, which differ from the predominantly aluminosilicate network structure of low-calcium geopolymers [9,10,11,12]. These binder systems have been considered promising alternatives to Portland cement because of their high early strength, low permeability, and resistance to aggressive environments. In recent years, alkali-activated materials have also been investigated for use in 3D printable concrete technologies [13].

A development that further advances the sustainability potential of alkali-activated concretes is the use of these binder systems in conjunction with industrial and urban waste [14]. Construction and demolition activities generate approximately 850 million tons of waste annually in the European Union and over 600 million tons in the USA. A significant portion of this waste consists of concrete and masonry materials. Recovering such waste as aggregate in concrete production both reduces natural resource consumption and alleviates pressure on landfills [15]. In this framework, recycled concrete aggregate (RCA) and waste glass aggregate (WGA) are among the two most studied waste material groups over the last decade [7,16,17].

The effects of RCA on concrete performance stem largely from the adhered mortar in its structure. Adhered mortar significantly increases the porosity and water absorption capacity of RCA compared to natural aggregate, while also causing the interfacial transition zone (ITZ) between the aggregate and matrix to be mechanically weaker [18]. Xiao et al. [19] reported that significant decreases in concrete compressive strength are observed when the RCA substitution ratio exceeds 30%. Similarly, Silva et al. [15] stated that RCA can be used safely up to certain ratios (usually 20–40%) with appropriate mixture design and curing conditions, but intolerable losses in strength may occur if these ratios are exceeded. In geopolymer systems, however, there are findings suggesting that alkaline activators may improve the ITZ by partially reacting with the adhered mortar on the RCA surface [14]. This suggests that geopolymer binders may have an advantage over Portland cement in limiting performance losses resulting from the use of RCA.

Waste glass aggregate presents a different set of problems. Approximately 130 million tons of glass waste is generated worldwide annually, but only 30–40% of this amount can enter recycling systems [20,21]. The biggest obstacle to using glass aggregate in concrete is the formation of a weak ITZ with the binder matrix due to its smooth surface and the risk of alkali-silica reaction (ASR) at high substitution ratios [22]. Kou et al. [23] reported that glass aggregate could be substituted for natural aggregate up to 20–25%, above which problems arise in both strength and ASR-induced expansion. Olofinnade et al. [16] noted that the ASR behavior of glass aggregate in geopolymer systems is more limited than in Portland cement systems, but strength losses due to low aggregate-matrix adhesion are similarly observed. Although the low water absorption capacity of glass aggregate is sometimes considered an advantage, it has also been reported that its smooth surface geometry and angular particle shape may prevent homogeneous distribution within the matrix, causing local stress concentrations [24].

At this point, fiber reinforcement comes into play to mitigate the negative effects of waste-derived aggregates on concrete performance. Fiber reinforcement can improve tensile and flexural performance and provide a more ductile fracture behavior by controlling crack propagation [25,26]. Sustainability-oriented studies on cement-based materials are not limited to geopolymer binders or recycled aggregates. Waste-derived fibers have also been investigated as alternative constituents in cementitious composites. For example, Bencardino et al. [27] evaluated cement-based mortars incorporating cellulose fibers derived from waste paper sludge and reported that these fibers could be used in mortar production while maintaining acceptable mechanical performance. These findings indicate that waste-derived fibers can contribute to resource efficiency and circular material use in cement-based composites. The bridging effect of fibers within the matrix delays or completely prevents the transformation of micro-cracks into macro-cracks [28].

In this way, fiber reinforcement can play a critical role in maintaining the overall mechanical integrity of the composite, especially in systems with weak ITZ properties (such as RCA or glass aggregate) [29,30]. The type, length, ratio, and distribution of the fibers, along with fiber-matrix adhesion, are the primary parameters determining the degree of this restorative effect [31,32].

In recent years, the concept of sustainability has also become influential in fiber reinforcement. Utilizing fibers obtained from waste streams instead of traditionally used steel, polypropylene, or carbon fibers is an approach aligned with circular economy principles. In this context, tire-derived textile fibers (TDTF), which remain after steel wires are separated in waste tire recycling facilities, have significant potential [33,34,35]. Every year, approximately 1.5 billion end-of-life tires emerge worldwide, and significant amounts of textile fibers are obtained as a by-product during their recycling. These fibers generally consist of a mixture of polyester, nylon, and polyamide, and can contribute to crack control and ductility within the concrete matrix [36]. The use of TDTF fibers in concrete and cement-based composites has shown meaningful improvements, especially in terms of flexural strength and toughness [31]. However, there are almost no studies on geopolymer systems where TDTF fibers are used together with aggregates having a weak ITZ, such as RCA and glass aggregate. It is also stated in the literature that fiber-reinforced alkali-activated concretes may exhibit different behaviors compared to traditional concretes in terms of microstructure and mechanical performance [37,38].

Previous studies have provided useful information on the use of RCA in concrete and alkali-activated systems, mainly focusing on the effects of adhered mortar, increased water absorption, and weaker interfacial transition zones [14,15]. Waste glass aggregate has also been investigated as a replacement material, with most studies emphasizing its smooth surface texture, low water absorption, and possible ASR-related concerns [16,23,24]. Separately, fiber reinforcement has been used to improve crack control and post-cracking behavior in cementitious and alkali-activated composites [25,26,29,30,31,32]. Tire-derived fibers, in particular, have been examined as waste-based reinforcement materials because of their potential contribution to ductility and crack bridging [31,33,34,35,36].

Although these studies provide an important basis, the combined use of RCA, waste glass aggregate, and TDTF in slag-rich alkali-activated concrete has not been sufficiently clarified. Previous reviews and experimental studies indicate that research on recycled aggregates in alkali-activated concretes has generally focused on a single recycled aggregate type or has not considered waste-derived fiber reinforcement together with RCA and waste glass aggregate [14,15,16]. Therefore, the present study compares RCA and waste glass aggregate at different volume replacement levels, both with and without TDTF reinforcement, within the same experimental program. This design allows the effects of aggregate type, replacement level, and fiber addition to be evaluated comparatively in terms of compressive strength, water absorption, and UPV (Table 1).

The present study addresses the combined use of recycled concrete aggregate, waste glass aggregate, and tire-derived textile fibers in geopolymer concrete. Previous studies have mainly examined these waste-derived materials separately or with a single recycled aggregate type. In contrast, this study compares two recycled aggregate types at different replacement levels, both with and without tire-derived textile fiber reinforcement, under the same experimental conditions. This approach allows the effects of aggregate type, replacement level, and fiber addition to be evaluated within a single mixture framework. The findings may contribute to mixture design strategies aimed at increasing waste utilization and reducing natural aggregate consumption in geopolymer concrete production.

## 2. Materials and Methods

### 2.1. Materials

Ground granulated blast-furnace slag (GGBS) was used as the primary reactive binder component. The GGBS was supplied by Bolu Oyak Cement, located in Bolu, Türkiye. Silica fume (SF) was incorporated at 15% by weight of the binder as a supplementary silica-rich component. In the present binder system, SF was not used as the main aluminosilicate precursor; instead, it was included to improve particle packing, support matrix densification, and contribute to the development of a more compact alkali-activated binder structure. The 15% SF content was selected based on preliminary mortar trials, in which SF contents above this level reduced workability and early-age compressive strength. The chemical compositions and physical properties of the GGBS and SF, as provided by the manufacturer, are presented in Table 2. Considering the high CaO content of GGBFS and the use of NaOH-Na_2_SiO_3_ activation, the mixtures produced in this study are classified as slag-rich alkali-activated concretes. The term geopolymer concrete is retained in the manuscript in line with common usage in the literature, but the binder chemistry corresponds to a calcium-rich alkali-activated slag-based system.

To initiate the geopolymerization process, a binary alkaline activator solution, prepared by mixing sodium hydroxide (NaOH) and sodium silicate (Na_2_SiO_3_) at specific ratios, was utilized. NaOH and Na_2_SiO_3_ with a purity of 98% were procured by Zag Kimya, located in İstanbul, Türkiye. The silica modulus (M_s_ = SiO_2_/Na_2_O) of the activator solution was fixed at 1.2, and the total Na_2_O content was taken as 6% by weight of the binder (GGBS + SF). The silica modulus (Ms = SiO_2_/Na_2_O) of the activator solution was fixed at 1.2, and the total Na_2_O content was set at 6% by weight of the binder (GGBS + SF). These values were selected based on preliminary mortar trials conducted with the same binder system and were also consistent with the ranges reported for alkali-activated slag mortars [49,50,51]. In the preliminary trials, Ms values of 1.0 and 1.2 and Na_2_O contents of 4% and 6% by binder weight were compared. The combination of Ms = 1.2 and 6% Na_2_O provided the highest 7-day compressive strength among the tested activator combinations, and this activator composition was therefore used in the concrete mixtures. The physical and chemical properties of the activator components are provided in Table 3 and Table 4.

Four different aggregates were utilized in this study: No. 1 crushed stone (natural coarse aggregate, D_max_: 12 mm), No. 2 crushed stone (natural fine aggregate, D_max_: 4 mm), waste glass aggregate, and recycled concrete aggregate (RCA). Aggregate replacement was performed on a volume basis. The waste glass aggregate was supplied by Bursa Cameks, located in Bursa, Türkiye, and its particle size was adjusted to the range of 4–11.2 mm. The RCA was obtained by crushing and sieving concrete waste collected from Bursa Metropolitan Municipality, Türkiye. The particle size distribution of all aggregates was determined via sieve analysis in accordance with the [52] standard, and the results are presented in Table 5. The gradations of the waste glass aggregate and RCA were not adjusted to exactly match that of the natural coarse aggregate. Accordingly, although the replacement was performed on a volume basis, the overall particle size distribution of the combined aggregate system may have changed depending on the recycled aggregate type and replacement level. The water absorption rates of the aggregates were determined according to [53] and were measured as 0.67% for No. 1 crushed stone, 0.40% for No. 2 crushed stone, 0% for waste glass aggregate, and 4.38% for RCA. The high water absorption capacity of RCA is attributed to the attached mortar in its structure [54,55]. Before mixing, the recycled concrete aggregates were immersed in water and brought to a saturated surface-dry (SSD) condition. This procedure was applied to reduce the influence of RCA absorption on the effective water content and water-to-binder ratio of the mixtures; therefore, no additional water compensation was used during mixing.

**Figure 1:**
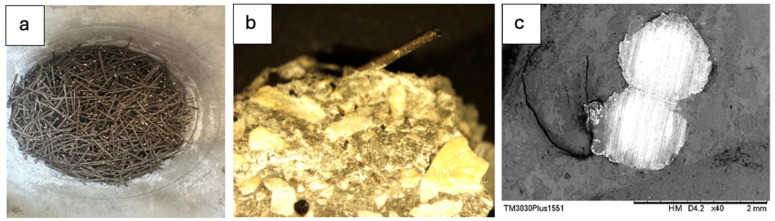
Tire-derived textile fibers used in the study: (**a**) fibers after preparation to the target length, (**b**) optical microscope image, and (**c**) SEM image.

In this study, textile fibers obtained from the waste tire recycling process (tire-derived textile fibers, TDTF) were used as reinforcement (Table 6). The fibers were supplied by a recycling facility and were recovered from end-of-life tires after magnetic separation of the steel fraction. The recovered fiber fraction mainly consisted of polyester-, nylon-, and polyamide-based fibers and also contained residual rubber or carbon-containing surface impurities. To provide direct characterization of the material used in this study, the fibers were examined in terms of geometry, density, and surface morphology. The main fiber properties are summarized in Table 6, and representative physical, optical microscope, and SEM images are presented in Figure 1. In all mixtures, the fiber content was fixed at 1% by volume.

### 2.2. Preparation of Mixtures

**Figure 2:**
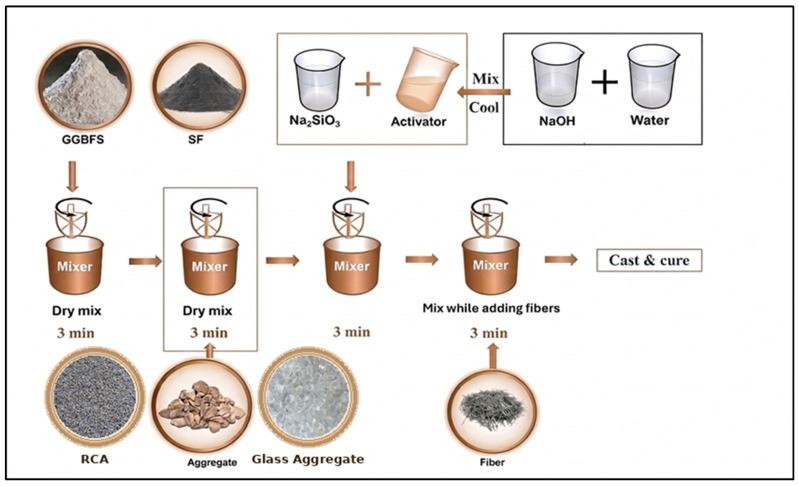
Preparation stage of geopolymer concrete mixtures.

A total of 22 different geopolymer concrete mixtures were prepared within the scope of this study (Figure 2). In these mixtures, the binder (GGBFS + SF) content was kept constant at 600 kg/m^3^, and the water/binder ratio was fixed at 0.29.

Waste glass aggregate and RCA were used to replace No. 1 crushed stone, selected as the natural coarse aggregate, at replacement levels of 20%, 40%, 60%, 80%, and 100% by volume. The corresponding mass quantities were calculated using the specific gravity of each aggregate and are presented in Table 7. For each replacement ratio, two series were produced: a control mixture without fibers and a mixture containing 1% TDTF (Tire-Derived Textile Fiber) with a length of 15 mm. Additionally, control mixtures containing only natural aggregates (both with and without fibers) were produced.

In all mixtures, the slump value was adjusted according to the S4 class (160–210 mm). The amount of material required for the production of 1 m^3^ of concrete is provided in Table 7. The mixture nomenclature was defined as ‘aggregate type–replacement ratio–fiber presence’. For example, ‘RCA-20-RF’ represents a mixture containing 20% recycled concrete aggregate and waste fibers (TDTF). ‘C’ denotes the control mixture, while ‘G’ refers to glass aggregate.

The alkali activator solution was prepared 24 h before the mixing process and kept in a closed container at room temperature (20 ± 2 °C). After NaOH pellets were dissolved in a quantity of distilled water, they were mixed with Na_2_SiO_3_ to obtain a homogeneous solution.

The mixture preparation process was carried out using a pan-type mixer with a 100 L capacity. Dry aggregates and binder materials (GGBFS and SF) were first mixed dry for 2 min. Then, the alkali activator solution was added, and mixing was continued for 3 min. TDTF fibers were gradually sprinkled onto the wet mixture to prevent clumping and ensure homogeneous distribution within the matrix, and an additional 2-min mixing process was applied. The total mixing time was approximately 7 min.

In specimen production, cube molds with dimensions of 100 × 100 × 100 mm were used. A total of 6 cube specimens were produced from each mixture, including three specimens for each testing age (7 and 28 days). The specimens were placed in the molds in accordance with the standards and then kept in their molds for 24 h. After the demolding process, the specimens were cured in a laboratory environment with a temperature of 20 ± 2 °C and a relative humidity of 65% ± 5 until the day of the experiment.

### 2.3. Test Procedures

**Figure 3:**
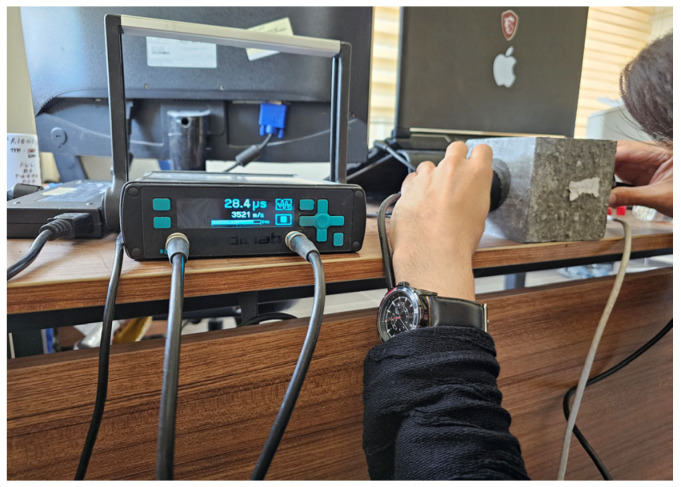
UPV test.

**Figure 4:**
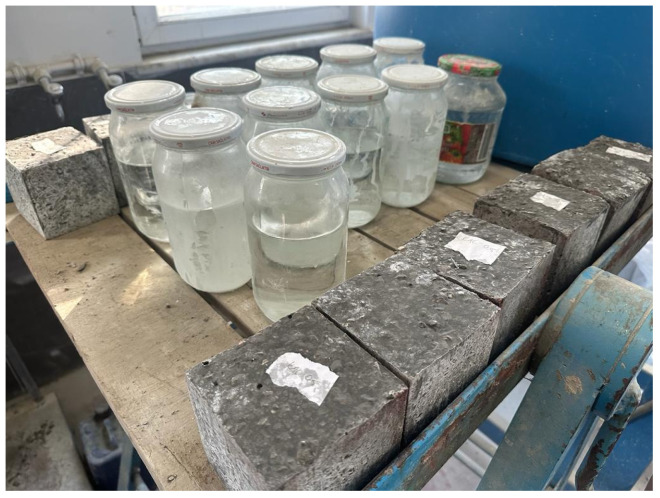
Demolded specimens.

**Figure 5:**
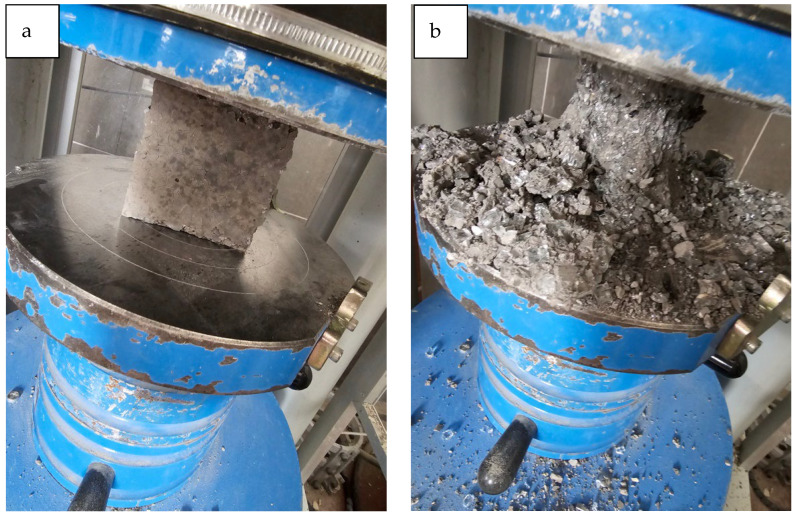
Representation of the specimen (**a**) before and (**b**) after being subjected to the compressive strength test.

The workability of the mixtures was evaluated by the slump test in accordance with the [56] standard. The slump values of all mixtures were controlled to be in the range of 160–210 mm (S4 class). The compressive strength, water absorption values, and ultrasonic pulse velocity (UPV) of the hardened specimens were determined according to [57,58,59] standards, respectively. The water absorption capacities by weight of the 28-day hardened concrete specimens were determined on 100 mm cube specimens. The experiments were carried out in a compression testing machine with a capacity of 3000 kN, with a loading rate of 0.5 MPa/s. Figure 3 shows the visual of the UPV test and Figure 4 shows the visuals of the specimens. In addition, Figure 5 shows the status of the specimens before and after being subjected to the compressive strength test.

## 3. Results and Discussion

### 3.1. Compressive Strength

The 7 and 28-day compressive strength values of the 22 different geopolymer concrete mixtures produced within the scope of the study are shown in Table 8. Looking at the table, the first impression is that waste fibers increase the strength under all conditions. While the 7-day strength of the control mixture was 49.1 MPa, this value increased to 61 MPa in its fibrous counterpart C-RF. At 28 days, an increase from 53.1 MPa to 67.2 MPa was experienced. Although the magnitude of this increase changed when waste aggregates were introduced into the mixture, the positive contribution of the fibers did not disappear in any mixture.

**Figure 6:**
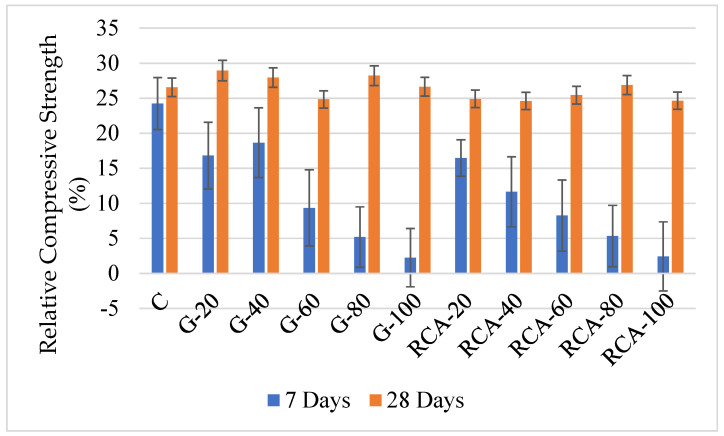
Effect of fiber usage on the relative strength values of geopolymer mixtures.

The effect of fiber usage on the relative strength values of geopolymer concrete mixtures is shown in Figure 6. As can be understood from the figure, the use of waste fibers positively affected the compressive strength performance of all geopolymer concrete mixtures. This positive effect became more dominant in 28-day specimens and was less affected by aggregate type and substitution ratio. This situation is explained by the fibers limiting the formation of micro-cracks within the concrete matrix and delaying crack propagation [60,61,62]. It has also been reported by various researchers that fiber usage increases load transfer capacity by partially improving weak interfacial regions and can improve the brittleness, crack resistance, flexural strength, and toughness of geopolymer concrete [63,64,65]. However, the positive effect of fiber usage on early-age strength values showed a decrease depending on the increase in waste glass and recycled concrete aggregate content. In geopolymer concrete mixtures containing glass aggregate, the relative strength values decreased from 16.8% to 2.3% with the increase in aggregate content. For mixtures containing recycled concrete aggregate, these values were calculated as 16.5% and 2.4%, respectively. The increase in the strength of geopolymer concrete mixtures over time has positively affected the working performance of waste fibers.

**Figure 7:**
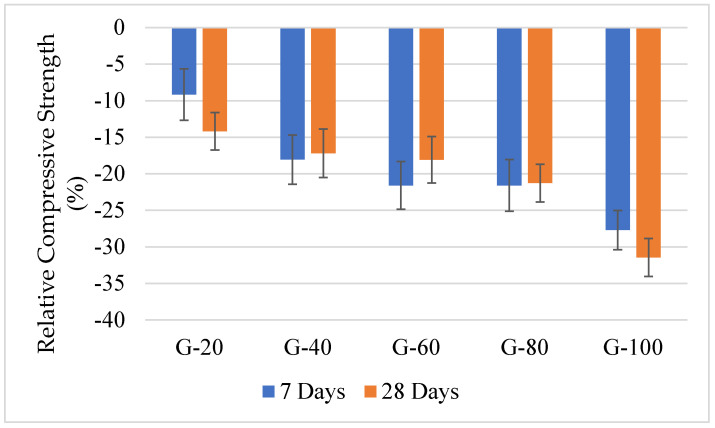
Effect of glass aggregate usage on strength compared to the control mixture.

In mixtures containing glass aggregate, it was observed that compressive strength decreased as the substitution ratio increased (Figure 7). This decrease ranged between 10–27% in 7-day specimens, while it occurred between 16–31% in 28-day specimens. The size, shape, and surface texture of aggregates have a significant impact on the strength performance of concrete.

Kuri et al. [66] determined that the use of recycled glass coarse aggregate reduced the compressive strength of geopolymer concrete by 11–26% and the tensile strength by 11–29%. This situation was attributed to the smooth surface of the glass aggregates and the formation of a weak bond with the binder matrix. Additionally, it was reported that the poor geometry of glass aggregates may have prevented the homogeneous distribution of the aggregates. Tahwia et al. [67] reported that using glass aggregate at a ratio of 22.5% reduced the compressive strength of fiber-free geopolymer concrete mixtures from 94.2 MPa to 88.1 MPa, and the strength of fiber-reinforced geopolymer concrete mixtures from 126 MPa to 121 MPa. Researchers explained this by the formation of an insufficient bond between the glass aggregate and the geopolymer matrix due to the smoothness of the glass surface. Furthermore, it was reported that the angularity of glass particles could create internal voids that negatively affect the development of compressive strength.

**Figure 8:**
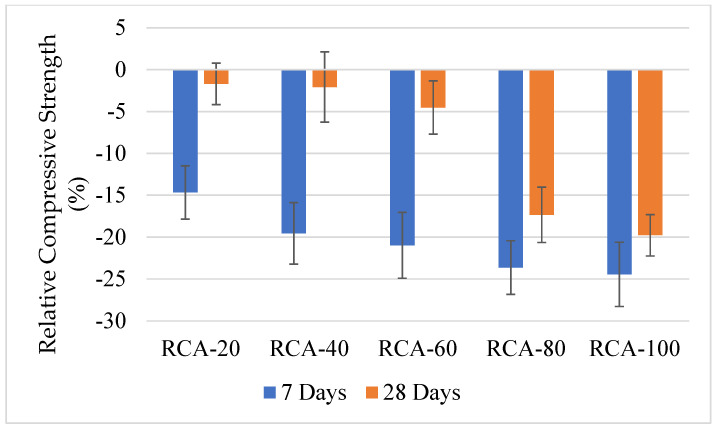
Effect of recycled concrete aggregate usage on strength compared to the control mixture.

The use of recycled concrete aggregate also reduced the compressive strength performance of geopolymer concrete mixtures compared to the control mixture. The decrease in 7-day specimens was calculated to be between 14–24% (Figure 8). Nuaklong et al. [68] reported that the compressive strength of geopolymer concrete containing recycled aggregate decreased by an average of 87%. This decrease was attributed to the low abrasion resistance resulting from the weakness of the adhered mortar in the recycled concrete aggregate [69]. Furthermore, it has been stated that adhered mortar has lower strength than conventional aggregate and new paste [70]. Similarly, Sata et al. and Nuaklong et al. [71,72] pointed to similar findings, attributing the strength decrease to the lower properties and high water absorption values of recycled concrete aggregate compared to natural aggregate. It was reported that the main factor affecting this reduction in strength is the weak interfacial transition zone between the old mortar and the new matrix [73,74]. A similar trend was also noted by [75]. However, a different picture emerges when the 28-day results are examined. With the use of recycled concrete aggregate up to a ratio of 60%, the decrease in compressive strength remained between 1–4%, while at higher substitution ratios, this decrease reached 17–19%.

When the geopolymer concrete series containing glass and recycled concrete aggregate were compared, the mixtures containing glass aggregate generally had higher strength values in the 7-day specimens. However, in the 28-day specimens, mixtures containing recycled concrete aggregate exhibited 3–17% higher strength. Kanagaraj et al. [76] also found that the contribution of recycled concrete aggregate was more positive according to the 28-day compressive strength results. Even at a 40% substitution level, it was observed that the substituted aggregates had a better bond with the binder. Additionally, the existing mortar content, particle size distribution, and the strength of the mortar in the recycled concrete aggregate were reported as factors responsible for strength development. However, significant strength losses occurred at high substitution ratios in both series. When the effect of waste fiber additive was evaluated, it was determined that it increased strength in both aggregate types but was particularly more effective in mixtures containing recycled concrete aggregate. This was explained by the fact that the capacity of the fibers to reinforce the weak microstructure became more pronounced. In addition to aggregate type and surface characteristics, differences in particle size distribution may also have contributed to the compressive strength results. The RCA used in this study had a finer and more continuous grading than the natural coarse aggregate, which may have improved packing density at moderate replacement levels and partly contributed to the limited 28-day strength loss observed up to 60% RCA replacement. In contrast, the waste glass aggregate had a different grading and a smoother surface texture, which may have reduced mechanical interlocking and weakened the aggregate–matrix bond. Therefore, the observed strength differences should be interpreted as the combined effect of aggregate type, replacement level, surface texture, water absorption capacity, and grading differences.

### 3.2. Water Absorption and UPV

**Figure 9:**
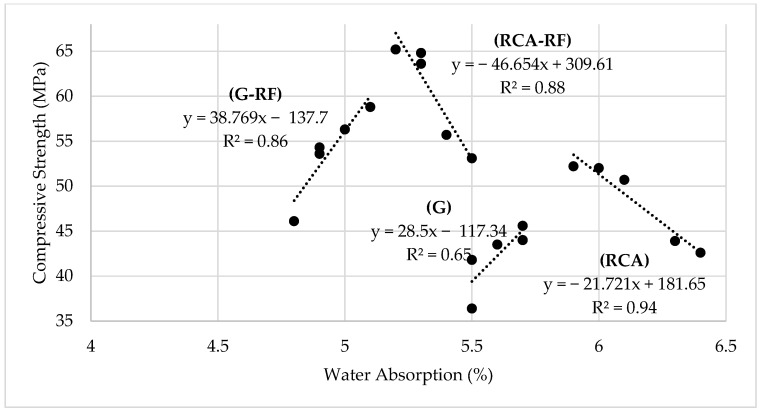
Relationship between compressive strength and water absorption for each mixture group.

The water absorption rates and UPV values of the 22 different geopolymer concrete mixtures produced within the scope of the study are shown in Table 9. When the water absorption capacities of the said mixtures were examined, it was understood that the addition of fibers significantly reduced the water absorption rates in all mixtures. For example, in the G-100 mixture, the water absorption value, which was 5.5% in the fiber-free specimen, decreased to the lowest value of 4.8% in the fibrous G-100-RF specimen. As the substitution ratio of glass (G) aggregate mixtures increased, the water absorption value decreased; conversely, the use of recycled aggregate (RCA) increased water absorption rates up to levels of 6.4%. This situation reveals that the high porosity and old mortar residues in the natural structure of RCA negatively affect the impermeability performance of the geopolymer system [69]. The correlation between the water absorption values and compressive strength values of the mixtures produced within the scope of the study is shown in Figure 9. To improve the clarity of the correlation analysis, the data were evaluated separately for each mixture group rather than using a single regression for all mixtures. This presentation allows the influence of aggregate type and fiber addition on the relationships between compressive strength, water absorption, and UPV to be compared more clearly.

When the graph is examined, both the lowest water absorption rates and high compressive strength were observed in the G-RF mixtures. The highest strength was obtained in RCA-RF mixtures. Independent of fiber substitution, a higher correlation exists in mixtures containing RCA, while a lower correlation exists in glass mixtures. Fiber substitution increased the correlation between compressive strength and water absorption rate. This situation was thought to result from fiber substitution transforming the void structure of the concrete into a more stable form by restricting capillary void continuity within the matrix and bridging micro-cracks [8]. This situation strengthened the correlation between the two parameters by allowing the physical mechanisms between compressive strength and water absorption rate to act more harmoniously with each other.

It is observed that the correlation (R^2^ = 0.65) is the lowest in the fiber-free G series mixtures with glass aggregate. This deviation may result from the change in the bond (ITZ) characteristics between the matrix and aggregate due to the smooth surface structure of the glass. 

It was observed that while the fiber additive negatively affected the compressive strength–UPV correlation, the compressive strength–water absorption rate correlation was positively affected. In fiber-free groups, both the water absorption–pressure and UPV–strength relationships were found to be very strong (r > 0.90). In fiber-free RCA mixtures, both water absorption and UPV showed an excellent linear relationship.

**Figure 10:**
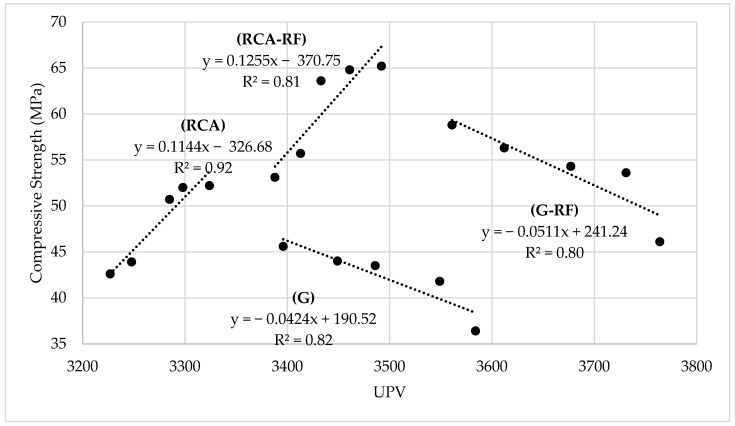
Relationship between compressive strength and UPV for each mixture group.

In the evaluation made over ultrasonic pulse velocity (UPV) values, a direct correlation was identified between the internal structure continuity of the specimens and fiber reinforcement. It was understood that specimens containing fibers had higher UPV values compared to fiber-free mixtures. The highest UPV value was obtained in the G-100-RF mixture with 3764 m/s, and the lowest UPV value was obtained in the fiber-free RCA-100 mixture with 3227 m/s. It is thought that the smooth surface structure of glass aggregates supports the sound velocity in a positive direction, whereas the structural discontinuities caused by the use of RCA block the propagation of sound waves, lowering the velocity values. In these mixtures, when compressive strength and water absorption values are compared, an inverse relationship between these two variables is clearly identified. Dense mixtures such as C-RF and G-100-RF, which had the lowest water absorption capacity, were also the specimens that exhibited the highest performance in compressive strength tests. The increase of water absorption to 6.4% in the RCA series decreased the compressive strength values to the range of 40–50 MPa. Fiber reinforcement compensated for this loss by increasing the mechanical strength by 20–30% through decreasing water absorption. The change in the relationship between the compressive strength of the concrete and the UPV value depending on the mixture groups is shown in Figure 10. Among the experimental groups, the fiber-free RCA group stands out with the highest correlation coefficient (R^2^ = 0.92). In contrast, although adding fibers to the mixture significantly increases the compressive strength, it causes the correlation to recede to levels of 0.81, as seen in the fibrous RCA group. Although adding fibers to the mixture provides a significant increase in compressive strength through the crack bridging mechanism, the fact that this mechanical improvement is not reflected in UPV values at the same rate caused the correlation to weaken.

**Figure 11:**
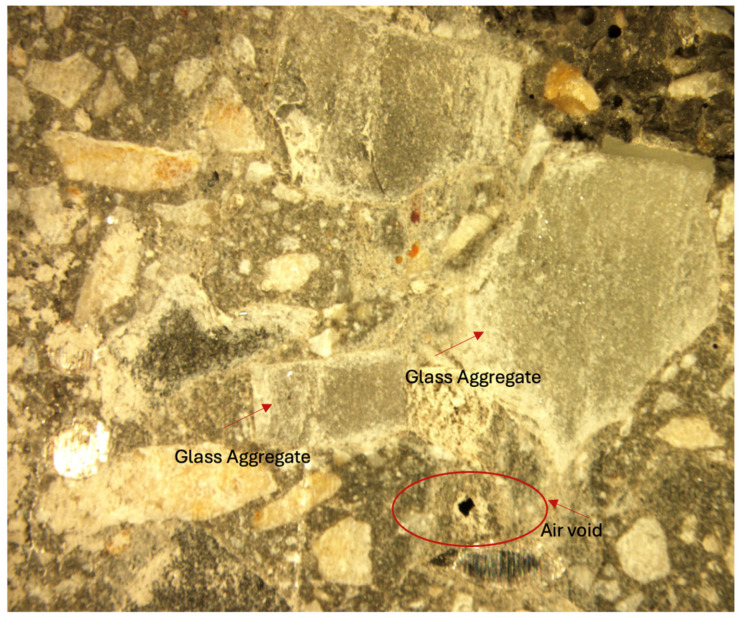
Optical microscope image of the glass aggregate surface.

In contrast to the RCA-containing mixtures, the G and G-RF series showed a negative relationship between UPV and compressive strength. This trend should be interpreted with caution. A possible explanation is that the smooth surface texture of the glass particles may allow relatively efficient ultrasonic wave transmission, while the same surface characteristics may limit mechanical interlocking with the geopolymer matrix. Therefore, higher UPV values in glass-containing mixtures do not necessarily indicate higher compressive strength. Since no direct microstructural bond analysis was performed in this study, this explanation is presented only as a possible interpretation of the observed trend (Figure 11). 

## 4. Conclusions

This study examined the combined use of waste glass aggregate, recycled concrete aggregate (RCA), and tire-derived textile fibers (TDTF) in geopolymer concrete. The results obtained from the 22 mixtures led to the following conclusions:

1. The addition of TDTF increased the compressive strength of all mixtures. This improvement was clearer at 28 days than at 7 days. In general, the 28-day strength increase provided by TDTF was approximately 25–30%, depending on the aggregate type and replacement level.
2. The effect of waste glass aggregate on compressive strength was more pronounced than that of RCA. Increasing the glass aggregate content caused a 16–31% reduction in 28-day compressive strength compared with the control mixture. This reduction was mainly related to the smooth surface of the glass particles and the weaker bond formed with the geopolymer matrix.
3. RCA showed a more favorable performance at moderate replacement levels. Up to 60% RCA replacement, the 28-day compressive strength loss remained limited to 1–4%. When the RCA content was increased to 80% and 100%, the strength loss reached 17–19%, indicating that high RCA contents were less suitable for maintaining strength.
4. The two recycled aggregates showed different behavior with curing age. Glass aggregate mixtures generally had higher 7-day strength, whereas RCA mixtures gave better results at 28 days. This difference can be related to the surface characteristics, adhered mortar content, water absorption capacity, and grading of the aggregates.
5. Water absorption results also differed between the two aggregate types. Glass aggregate mixtures had lower water absorption values, especially when TDTF was used. In contrast, RCA mixtures showed higher water absorption because of the porous adhered mortar remaining on the RCA particles.
6. The UPV results did not show the same trend for all mixtures. RCA-containing mixtures generally showed a positive relation between UPV and compressive strength. In the glass aggregate mixtures, however, higher UPV values did not always correspond to higher compressive strength. This may be associated with the smooth surface of glass particles, although direct microstructural evidence would be needed to confirm this explanation.
7. Considering compressive strength, water absorption, and UPV together, RCA replacement up to 60% and glass aggregate replacement between 40% and 60% gave the most balanced results among the mixtures tested. These levels should be interpreted for the present materials and mixture design, rather than as general limits for all geopolymer concretes.

Further studies should include flexural strength, splitting tensile strength, toughness, fracture energy, durability tests, and microstructural observations to evaluate the contribution of TDTF more completely. Since these tests were not included in the present experimental programme, the beneficial effect of TDTF reported in this study should be interpreted only in terms of compressive strength, water absorption, and UPV.

## Figures and Tables

**Table 1:** Waste-derived aggregates and fiber used in selected geopolymer concrete studies.

Study	Fiber Type	Aggregate Type	Highlights
[39]	Waste Steel Wire (WSW)	No waste aggregate	WSW (0–3%) increased compressive strength by 8.5–24.9% under ambient curing. Maximum compressive strength reached 42.56 MPa under steam curing. Flexural strength improved by 33.8% with 3% WSW. 20% GGBS + 3% WSW provided sufficient strength without steam curing.
[40]	Waste Tire Steel Fiber (WTSF) & Hemp Fiber (HF)	Recycled Fine Aggregate (RFA) & Waste Marble Powder	WTSF increased compressive and flexural strength by 42%. WTSF improved fracture energy by 840%. Hybrid fibers (WTSF + HF) increased strength by 21% and fracture energy fourfold. HF burned at 600 °C; WTSF experienced corrosion during freeze–thaw cycles.
[41]	Waste Tire Steel Fiber (WTSF)	Crumb Rubber (5%, 10%, 15% replacement for fine aggregate)	10% rubber waste significantly reduced workability and compressive strength, while other mechanical properties decreased by 6–10%. WTSF reinforcement compensated for performance by increasing compressive strength up to 90% and tensile strength up to 42%. Combined use of rubber and fibers increased energy absorption capacity up to 312%, enhancing impact resistance.
[42]	Steel, Polyamide, & Polyethylene Fibers	Recycled Concrete Aggregate (RCA) & Glass Powder (GP)	50% replacement of RCA with GP yielded the best strength results. Steel fiber increased flexural strength by 42.9% at 900 °C. Steel fiber showed the best performance in high-temperature and freeze–thaw resistance. A metakaolin-red mud based matrix was utilized.
[43]	Waste Tire Wire (RSW) & Polyvinyl Alcohol (PVA) Fiber	One-part geopolymer matrix (Powder sodium metasilicate)	Steel fibers increased compressive strength by up to 18%, while PVA fibers decreased it by up to 14%. The number of fibers per unit area (Ns) was identified as the key factor determining post-crack load-carrying capacity. Hybrid RSW + PVA provided significant improvements in ductility index and toughness.
[44]	Waste Steel Fiber	Recycled Coarse Aggregate (RCA)	5.71% higher compressive strength was achieved with fiber use compared to the control. Fiber content exceeding 5% negatively affected mechanical properties. ITZ was identified as the weakest point where failure initiated under compression. The developed stress–strain model aligned with experimental data for structural behavior prediction.
[45]	No Fiber	RCA & Crushed Glass Sand	RCA + glass sand combination reduced ASR risk. Glass sand doubled expansion, but 30% Fly Ash (FA) reduced this by 88%; GGBS had a lower effect on preventing expansion. Although glass sand significantly reduced strength, the 30% FA + 20% GGBS combination restored strength to control levels. Glass sand increased water absorption by 67% and weakened chloride resistance; however, 20% GGBS significantly improved it.
[46]	No Fiber	Crumb Rubber (CR), Waste Glass (GW), & Waste Ceramic (CW)	CW increased compressive strength significantly by 24–29% at 10% and 40% substitution levels; CR reduced strength by 69.2–83.5%. GW effectively reduced water absorption by 66–72% compared to CW and CR. Both CW and GW improved chemical resistance, reducing strength loss under sulfate and acid attacks by 15–33%.
[47]	Polypropylene Fiber (PPF) & Glass Fiber (GF)	Crumb Rubber	Hybrid PPF + GF reinforcement increased compressive strength by 198% and flexural strength by 261%. 1% PPF reduced thermal conductivity by 2.23%. GF exhibited superior durability under high temperature and mechanical stress.
[48]	Waste Tire Wire (WTW)	GGBS + Fly Ash binder	WTW (1%, 2%, 3%) and GGBS + SF (5%, 10%, 15% replacement) were tested. Strength increased by ~50% to reach 45 MPa under ambient curing. A maximum compressive strength of 65 MPa was achieved under steam curing.

**Table 2:** Physical and Chemical Properties of Ground Granulated Blast-Furnace Slag and Silica Fume.

Component/Property	Unit	GGBFS (Slag)	Silica Fume
SiO_2_	%	35.5	75.9
Al_2_O_3_	%	12.4	2.05
Fe_2_O_3_	%	1.5	1.07
CaO	%	38.9	0.763
MgO	%	5.05	10.9
SO_3_	%	1.67	0.96
Na_2_O	%	-	1.96
K_2_O	%	-	1.69
Cl	%	-	0.01
Loss on Ignition	%	1.08	2.3
Specific Gravity	Dimensionless	2.9	2.41

**Table 3:** Physical and Chemical Properties of Sodium Hydroxide.

Appearance	NaOH	Na_2_CO_3_	NaCl	Fe	Density
Colorless and Clear	98%	max 0.25%	max 0.02%	8 ppm	2.13 g/cm^3^

**Table 4:** Physical and Chemical Properties of Sodium Silicate.

Appearance	SiO_2_/Na_2_O	Na_2_O	SiO_2_	Cl	SO_4_	Density
Clear Viscous Liquid	2.13	11.27%	24.00%	0.01%	0.01%	1.44 g/cm^3^

**Table 5:** Particle Size Distribution of the Aggregates Used.

Sieve Size (mm)	Crushed Stone Aggregate No. 1 (%)	Crushed Stone Aggregate No. 2 (%)	Waste Glass Aggregate (%)	Recycled Concrete Aggregate (%)
0.063	3.2	0	0	0
0.25	8	0	0	0
0.5	17.3	0	0	0
1	30.4	0	0	0
2	59.7	0	0	0
4	97.9	0.8	2.3	3.9
5.6	99.6	7.8	7.2	8.6
8	100	50.6	65.6	72.9
11.2	100	100	100	100
16	100	100	100	100

**Table 6:** Properties of TDTF.

Properties	TDTF
Source	Waste Tire Fiber
Raw Material	Carbon Coated Aramid
Geometry	Straight
Length (mm)	15/30 mm
Diameter (mm)	0.60 mm
Fragility	25/50
Density (g/cm^3^)	1.86
Tensile Strength (MPa)	>540 MPa
Modulus of Elasticity	8 GPa
Surface Morphology	Rough and Carbon Residue

**Table 7:** Theoretical mix proportions for 1 m^3^ of GC (kg).

Mix	GGBFS	Silica Fume	Na_2_SiO_3_	NaOH	Water	Fine Agg.	Coarse Agg.	Waste Agg.	Fiber
C-RF	510	90	158.9	19.6	78.1	765.26	750.05	0	18.6
RCA-20-RF	510	90	158.9	19.6	78.1	765.26	600.04	133.78	18.6
RCA-40-RF	510	90	158.9	19.6	78.1	765.26	450.03	267.55	18.6
RCA-60-RF	510	90	158.9	19.6	78.1	765.26	300.02	401.33	18.6
RCA-80-RF	510	90	158.9	19.6	78.1	765.26	150.01	535.11	18.6
RCA-100-RF	510	90	158.9	19.6	78.1	765.26	0	668.89	18.6
G-20-RF	510	90	158.9	19.6	78.1	765.26	594.68	140.73	18.6
G-40-RF	510	90	158.9	19.6	78.1	765.26	444.67	281.47	18.6
G-60-RF	510	90	158.9	19.6	78.1	765.26	294.66	422.2	18.6
G-80-RF	510	90	158.9	19.6	78.1	765.26	144.65	562.94	18.6
G-100-RF	510	90	158.9	19.6	78.1	765.26	0	703.67	18.6
C	510	90	158.9	19.6	78.1	765.26	774.65	0	-
RCA-20	510	90	158.9	19.6	78.1	765.26	624.64	133.78	-
RCA-40	510	90	158.9	19.6	78.1	765.26	474.63	267.55	-
RCA-60	510	90	158.9	19.6	78.1	765.26	324.62	401.33	-
RCA-80	510	90	158.9	19.6	78.1	765.26	174.61	535.11	-
RCA-100	510	90	158.9	19.6	78.1	765.26	0	693.49	-
G-20	510	90	158.9	19.6	78.1	765.26	619.28	140.73	-
G-40	510	90	158.9	19.6	78.1	765.26	469.27	281.47	-
G-60	510	90	158.9	19.6	78.1	765.26	319.26	422.2	-
G-80	510	90	158.9	19.6	78.1	765.26	169.25	562.94	-
G-100	510	90	158.9	19.6	78.1	765.26	0	728.27	-

**Table 8:** 7 and 28-day compressive strength values of geopolymer mixtures.

Mixture	Compressive Strength (MPa)					
	7 Days			28 Days		
	Mean	Standard Deviation	Coefficient of Variation	Mean	Standard Deviation	Coefficient of Variation
C	49.1	3.1	6.2	53.1	2.3	4.3
C-RF	61	2.6	4.3	67.2	1.8	2.6
G-20	44.6	1.1	2.5	45.6	1.3	2.8
G-20-RF	52.1	1.1	2.2	58.8	3.4	5.8
G-40	40.2	1.4	3.4	44	2.4	5.4
G-40-RF	47.7	1.7	3.6	56.3	2.3	4.0
G-60	38.5	1.4	3.7	43.5	2.2	5.1
G-60-RF	42.1	2.3	5.4	54.3	3.0	5.5
G-80	38.5	1.8	4.7	41.8	1.5	3.7
G-80-RF	40.5	1.3	3.3	53.6	2.4	4.5
G-100	35.5	0.5	1.5	36.4	1.8	5.0
G-100-RF	36.3	1.5	4.1	46.1	1.3	2.8
RCA-20	41.9	0.7	1.7	52.2	0.2	0.3
RCA-20-RF	48.8	2.5	5.0	65.2	2.3	3.6
RCA-40	39.5	1.9	4.8	52	3.1	6.0
RCA-40-RF	44.1	1.2	2.8	64.8	2.3	3.5
RCA-60	38.8	2.3	5.9	50.7	1.9	3.8
RCA-60-RF	42	0.9	2.2	63.6	3.7	5.9
RCA-80	37.5	1.4	3.7	43.9	2.4	5.4
RCA-80-RF	39.5	1.1	2.8	55.7	1.4	2.5
RCA-100	37.1	2.3	6.2	42.6	1.3	3.1
RCA-100-RF	38	0.8	2.0	53.1	3.3	6.1

**Table 9:** Water absorption rate and UPV values of geopolymer mixtures.

Mixture	Water Absorption (%)			UPV (m/s)		
	Mean	Standard Deviation	Coefficient of Variation	Mean	Standard Deviation	Coefficient of Variation
C	5.80	0.20	3.44	3348	132.24	3.95
C-RF	5.10	0.27	5.30	3515	69.55	1.98
G-20	5.70	0.24	4.15	3396	159.00	4.68
G-20-RF	5.10	0.32	6.18	3561	151.62	4.26
G-40	5.70	0.26	4.48	3449	199.10	5.77
G-40-RF	5.00	0.24	4.84	3612	134.25	3.72
G-60	5.60	0.20	3.62	3486	84.33	2.42
G-60-RF	4.90	0.25	5.19	3677	208.15	5.66
G-80	5.50	0.27	4.98	3549	177.69	5.01
G-80-RF	4.90	0.16	3.34	3731	176.44	4.73
G-100	5.50	0.32	5.82	3584	127.17	3.55
G-100-RF	4.80	0.14	2.94	3764	116.72	3.10
RCA-20	5.90	0.36	6.02	3324	204.51	6.15
RCA-20-RF	5.20	0.30	5.82	3492	205.80	5.89
RCA-40	6.00	0.37	6.09	3298	153.93	4.67
RCA-40-RF	5.30	0.29	5.44	3461	115.76	3.34
RCA-60	6.10	0.33	5.45	3285	177.59	5.41
RCA-60-RF	5.30	0.09	1.63	3433	170.68	4.97
RCA-80	6.30	0.22	3.44	3248	201.59	6.21
RCA-80-RF	5.40	0.19	3.52	3413	197.33	5.78
RCA-100	6.40	0.24	3.72	3227	102.06	3.16
RCA-100-RF	5.50	0.27	4.95	3388	80.18	2.37

## Data Availability

The original contributions presented in this study are included in the article. Further inquiries can be directed to the corresponding author.
